# Fibroblast growth factor 1 ameliorates adipose tissue inflammation and systemic insulin resistance via enhancing adipocyte mTORC2/Rictor signal

**DOI:** 10.1111/jcmm.15872

**Published:** 2020-09-26

**Authors:** Longwei Zhao, Miaojuan Fan, Lijun Zhao, Hongyan Yun, Yan Yang, Chen Wang, Di Qin

**Affiliations:** ^1^ State Key Laboratory of Natural Medicines School of Life Science and Technology China Pharmaceutical University Nanjing China; ^2^ Maternal and Child Health Hospital of Zhuang Lang Pingliang China; ^3^ Foshan Chancheng Central Hospital Guangdong China; ^4^ School of Sports and Health Nanjing Sport Institute Nanjing China; ^5^ Jiangsu Sports and Health Engineering Collaborative Innovation Center Nanjing China

**Keywords:** adipocyte mTORC2/Rictor, adipose tissue macrophages, fibroblast growth factor 1, inflammation, insulin resistance, monocyte chemoattractant protein‐1/C‐C chemokine ligand 2

## Abstract

Obesity‐induced activation and proliferation of resident macrophages and infiltration of circulating monocytes in adipose tissues contribute to adipose tissue inflammation and insulin resistance. These effects further promote the development of metabolic syndromes, such as type 2 diabetes, which is one of the most prevalent health conditions severely threatening human health worldwide. Our study examined the potential molecular mechanism employed by fibroblast growth factor 1 (FGF1) to improve insulin sensitivity. The leptin receptor‐deficient obese mice (*db/db*) served as an insulin‐resistant model. Our results demonstrated that FGF1‐induced amelioration of insulin resistance in obese mice was related to the decreased levels of pro‐inflammatory adipose tissue macrophages (ATMs) and plasma inflammatory factors. We found that FGF1 enhanced the adipocyte *mTORC2/Rictor* signalling pathway to inhibit C‐C chemokine ligand 2 (CCL2) production, the major cause of circulating monocytes infiltration, activation and proliferation of resident macrophages in adipose tissues. Conversely, these alleviating effects of FGF1 were substantially abrogated in adipocytes with reduced expression of mTORC2/rictor. Furthermore, a model of adipocyte‐specific *mTORC2/Rictor*‐knockout (AdRiKO) obese mice was developed to further understand the in vitro result. Altogether, these results demonstrated adipocyte *mTORC2/Rictor* was a crucial target for FGF1 function on adipose tissue inflammation and insulin sensitivity.

## 1 INTRODUCTION

Obesity‐induced chronic tissue inflammation is generally considered to be the primary cause for the occurrence and development of insulin resistance and type 2 diabetes mellitus (T2DM).^1^ Results from basic research and clinical characterization suggest the inflammation of adipose tissues (especially epididymal white adipose tissue, eWAT) is the main cause of systemic inflammation and insulin resistance.^1^, ^2^ Therefore, effective mitigation of WAT inflammation is a crucial strategy for managing systemic insulin resistance in obesity.^1^, ^2^ Activation of adipose tissue macrophages (ATMs) usually plays a significant role in obesity‐induced inflammation and insulin resistance. Moreover, the subsequent macrophage‐induced pro‐inflammatory responses cause chronic inflammation in WAT.^3^, ^4^ Macrophage scavengers, such as clodronate liposomes, have, therefore, been applied to improve the metabolic profile by reducing the concentration of visceral adipose tissue macrophages in diet‐induced obese mice.^5^, ^6^

Macrophages are generally classified two groups, namely pro‐inflammatory macrophages (M1) and anti‐inflammatory macrophages (M2). M1 secrete pro‐inflammatory factors, such as *Tnf‐α* and *Il‐6*,^1^, ^7^ and express the surface marker *Cd11c*.^8^, ^9^ On the other hand, M2 produce anti‐inflammatory cytokines, such as *Il*‐4 and *Il*‐10, and express the surface marker*Cd206*.^9^ Thus, the disproportionate increase in WAT pro‐inflammatory macrophages during obesity causes adipose tissue inflammation.^1^, ^7^, ^8^, ^9^, ^10^ Two theories have been posited to explain the increase in M1 macrophages in WAT during obesity. One view is that bone marrow monocytes are recruited into WAT, where they differentiate into pro‐inflammatory macrophages.^9^, ^11^ The other is that obesity triggers the proliferation and activation of resident macrophages in WAT.^12^ In both theories, the monocyte chemoattractant protein 1/C‐C chemokine ligand 2 (CCL2) produced by adipocytes is presumably the predominant contributor to the increase in M1 macrophage levels in WAT.^12^, ^13^, ^14^ Therefore, the number of macrophages in WAT during obesity could be dependent on the expression of adipocyte *Ccl2*.^12^, ^13^, ^14^ Loss of *rictor*, a crucial component of mTORC2 that played a role in growth and metabolism,^15^ could lead to overexpression of *Ccl2* in the adipocytes.^16^ Therefore, adipocyte *mTORC2/Rictor* is a vital target for inhibiting the infiltration of circulating monocytes and the proliferation and activation of resident macrophages in the adipose tissue.

As an autocrine/paracrine regulator, fibroblast growth factor 1 (FGF1) is mitogenic on cells of various origins, including liver, vasculature, and skin.^17^ Therefore, this property has propelled the clinical application of recombinant human Fibroblast Growth Factor 1 (rhFGF1) peptides to facilitate wound/burn repair and ulcer regeneration for over a decade.^18^ Recent studies have reported the characteristic of insulin resistance in *FGF1* knockout mice fed on a high‐fat diet (HFD). In contrast, the administration of exogenous rhFGF1 significantly improved insulin sensitivity in T2DM, suggesting a potential role in nutrient homeostasis.^19^, ^20^ Moreover, the HFD‐fed *FGF1^−/−^* mice exhibited a significant increase in serum inflammatory factors, and chronic administration of rhFGF1 could reduce the levels of serum inflammatory cytokines in T2DM.^19^, ^20^ However, the precise signalling cascades related to adipose tissue inflammation and regulated by *FGF1* have not yet been elucidated, and the underlying mechanism is obscure. Therefore, the *FGF1*‐regulated signalling pathways involved in systemic inflammation, as well as the molecular mechanism by which FGF1 ameliorates insulin resistance, deserve further exploration.

Therefore, in the present study, we explored the possible molecular mechanism by which FGF1 ameliorates adipose inflammation and systemic insulin resistance. Our findings indicated that rhFGF1 significantly reduced pro‐inflammatory macrophage levels in adipose tissues by enhancing the *mTORC2/* *R* *ictor* signalling pathway to inhibit *C* *cl2* expression. Conversely, these alleviating effects of FGF1 were substantially abrogated in adipocytes with reduced *mTORC2/* *R* *ictor* signalling. Further, we developed a model that genetically induced the adipocytes‐specific *mTORC2/* *R* *ictor*‐knockout (AdRiKO) obese mice to understand the role of *mTORC2/* *R* *ictor* in mediating FGF1 on adipose tissue inflammation and insulin resistance. Thus, our study elucidated the molecular mechanism of FGF1 as an insulin sensitizer on the metabolic profile, and the effect of FGF1 on the ATMs infiltration and proliferation might represent an attractive therapeutic target for preventing the development of adipose tissue inflammation and insulin resistance.

## 2 MATERIAL AND METHODS

### 2.1 Protein expression and purification

FGF1 was expressed and purified, as described in a previous study.^21^

### 2.2 Animals and experiments

Male *db/db* mice of the *C57BL/6J* strain were purchased from the Model Animal Research Center of Nanjing University and fed on the standard chow diet. The adipose tissue‐specific *Rictor*‐knockout (AdRiKO) mice were described previously.^22^, ^23^, ^24^ All animal experiments were performed in accordance with the National Institutes of Health Guide for the Care and Use of Laboratory Animals and were approved by the Center for New Drug Evaluation and Research, China Pharmaceutical University, Nanjing, China.

Given the infertility of AdRiKO mice, *Rictor^flox/flox^* mice were bred with *Adiponectin‐cre^+/‐^* mice to generate mice for our experiments. Age‐matched *cre*‐negative males were used as controls. Animals were fed on HFD (60% kcal from fat) for 10 weeks (Research Diets Inc). All animals were maintained under a 12‐h light‐dark photoperiod at 22±°C (50%‐60% humidity, lights on at 7 am) and were allowed free access to food and water. The *db/db*, HFD and AdRiKO mice were randomly divided into two groups and treated with FGF1, while negative treatment control mice were treated with vehicle (PBS).

For the evaluation of chronic efficacy, *db/db* and AdRiKO mice were intraperitoneally injected with FGF1 at a dose of 0.5 mg/kg body weight or PBS every other day for 4 weeks, the administration dose of FGF1 was based on our previous study.^21^ The control groups were administrated with an equal volume of PBS at the same time. At the indicated time‐points, blood glucose level was assessed using a blood glucose meter and calibrated by following its instructions (Roche).

### 2.3 Evaluation of insulin sensitivity in vivo

We performed intraperitoneal glucose tolerance test (IPGTT) and Intraperitoneal insulin tolerance test (IPITT) to evaluate the effect of FGF1 on insulin sensitivity in obese mice after chronic treatment. Firstly, before IPGTT, mice were fasted overnight (6‐8 hours) and then challenged with a glucose solution (1.5 g/kg body weight, IP). The blood glucose level was then determined 0, 15, 30, 60, 90 and 120 minutes post‐injection as described above. Area under the curve (AUC) was calculated by the GraphPad Prism 7 software (GraphPad Software). For IPITT, mice were conducted after 3 hours of fasting and injected with human insulin (Humulin R, IP; Eli Lilly, Indianapolis, IN) at a dose of 1.5 unit/kg body weight. The blood glucose level was assessed 0, 15, 30, 60, 90 and 120 minutes after insulin injection. AUC was calculated by the Origin 7.5 software (Origin Lab Corporation). The plasma levels of CCL2, insulin and glucagon were evaluated using a commercial ELISA kit (R&D Systems) according to the manufacturer's instructions.

### 2.4 Pathological, histopathological and immunohistochemical evaluation

eWAT from healthy and obese mice were excised following chronic administration of PBS and FGF1, respectively. Tissues were fixed in 4% paraformaldehyde overnight and embedded in paraffin. For immunocytochemistry, 5 μm thick paraffin sections were incubated overnight with primary antibody (rabbit polyclonal to CD68 [1:500] and UCP‐1[1:500] from Abcam) at 4°C after deparaffinization. Thereafter, the sections were incubated with a horseradish peroxidase‐conjugated secondary antibody against rabbit (1:200), developed with a DAB (3, 3‐diaminobenzidine) developing system (Beyotime Biotechnology), counterstained with haematoxylin and observed under a light microscope.

Immunofluorescence staining of eWAT was performed as previously described.^25^ Briefly, the eWAT paraffin sections were incubated with primary antibody (rabbit polyclonal to F4/80 [1:500, Cat#: ab60343]; Armenian hamster monoclonal anti‐CD11c [1:500, Cat#: ab33483]; Armenian hamster monoclonal anti‐PCNA [1:900; Cat#: 18197], Abcam Cambridge, MA; and rabbit polyclonal to MCP‐1[1:500, Cat#: NBP1‐07035], Novus biological) overnight at 4°C after deparaffinization. They were then washed thrice with PBS. Finally, for immunofluorescence single staining, fluorochrome‐conjugated secondary antibody against a donkey anti‐rabbit IgG H&L (Alexa Fluor^®^ 488) (1:1000 dilution) (Abcam) was added to the sections and co‐incubated at room temperature for 1 hour. Tissue sections were imaged on an inverted confocal microscope (Leica SP8 X). For immunofluorescence double staining, fluorochrome‐conjugated secondary antibodies against a donkey anti‐rabbit IgG H&L (Alexa Fluor^®^ 488) (1:1000 dilution) (Abcam) and a goat anti‐Armenian hamster IgG H&L (Alexa Fluor^®^ 647) (1:1000 dilution) (Abcam) were added to the sections and co‐incubated at room temperature for 1 hour. Tissue sections were imaged on an inverted confocal microscope (Leica SP8 X).

### 2.5 3T3‐L1 adipocytes culture

3T3‐L1 pre‐adipocytes (3T3‐L1 MBX ATCC^®^ CRL‐3242™) were cultured as previously described^26^ and seeded in 6‐well plates at a density of 150 000 cells/well in 1 mL culture medium. The cells were differentiated into mature adipocytes, as previously described.^27^ Accumulation of lipid droplets was observed in >95% of cells after 14 days. Cells at day 14‐15 were used for experiments. Effect of mTORC2 on the mediation of insulin sensitivity was assayed by stimulating mature adipocytes with 100 ng/mL of FGF1^21^ and 100nM of insulin in the presence or absence of torin1 (250 nmol/L, a specific inhibitor of mTORC2).^16^ We subsequently measured the levels of CCL2 in adipocyte supernatant using a commercial ELISA kit (R&D Systems) following the manufacturer's instructions.

### 2.6 Western blot analysis

3T3‐L1 adipocytes or mouse tissues were lysed in RIPA lysis buffer (25 mmol/L Tris, pH 7.6, 150 mmol/L NaCl, 1% NP‐40, 1% sodium deoxycholate, 0.1% SDS), containing protease and phosphatase inhibitors (Thermo Fisher Scientific). The concentration of total protein in the samples was assessed using a BCA Kit (Protein Assay Kit, Beyotime Biotechnology). The proteins (40 µg) were separated on an 8%‐12% SDS‐PAGE and electro‐transferred onto a nitrocellulose membrane. Protein blots were probed with antibodies against CD68 (Abcam; Cat#: ab31630), CCL2 (Novus biological; Cat#: NBP1‐07035), Phospho‐IKKα/β (Cat#: ab59195), IKKα/β (Santa, Cat#: sc‐7607), Phospho‐IKBα (Santa, Cat#: sc‐8404) and IKBα (Santa, Cat#: sc‐371). The following antibodies used in this study were purchased from Cell Signaling Technology: GAPDH (Cat#: 51332); phospho‐AKT (ser473, Cat#: 4060); AKT (Cat#: 9272); RICTOR (Cat#: 2140); phospho‐JNK (Thr183/Tyr185, Cat#: 9255,); JNK (Cat#: 9252) and TNF‐α (Cat#: 3707s).

### 2.7 RNA extraction, cDNA synthesis and quantitative RT‐PCR

Total RNA was extracted from mouse eWAT using a Trizol reagent (Thermo Fisher Scientific) and reverse transcribed into complementary DNA (cDNA) with Prime Script RT reagent kit (Takara). Quantitative real‐time PCR was performed using SYBR Premix Ex Taq (Takara) with specific primers (listed in the supplemental Table S1) on a Step One Plus Real‐Time PCR system (Applied Biosystems^®^ Quant Studio^®^ 3). UBC was used as an endogenous control.

### 2.8 *In vitro* chemotaxis assay

*In vitro* chemotaxis assay was performed as previously described^28^ using the mouse‐derived macrophage cell line RAW264.7. For the migration, 100 000 macrophages were placed in the upper chamber of an 8‐μm polycarbonate filter (24‐Transwell format; Corning) after treatment with FGF1 (100 ng/mL) for 30 minutes. The specific inhibitor of CCR2 (INCB3344:100 nmol/L)^29^ and PBS was, respectively, used as positive and negative controls. The lower chamber contained either 3T3L1 differentiated adipocyte conditioned medium or DMEM complete medium were placed in the lower chamber. After 3 hours of migration, cells were fixed in formalin, stained with crystal violet, imaged and counted using the ImageJ software.

### 2.9 Proteome analysis

eWAT was isolated from the vehicle group and *db/db* mice treated with FGF1. Proteome analysis was performed as previously described.^16^, ^30^ Details on the experimental procedure are provided in the supplemental information section.

### 2.10 Statistical analysis

*In vitro* experiments were repeated in triplicate. The results were expressed as mean ± SEM. The charts in the figures were generated by software of GraphPad Prism 7. Statistical analysis was performed with one‐way ANOVA by using statistical software NASDAQ: SPSS from SPSS Inc *P* < .05 was considered to be statistically significant.

## 3 RESULTS

### 3.1 FGF1 ameliorates insulin resistance in *db/db* mice

**FIGURE 1:**
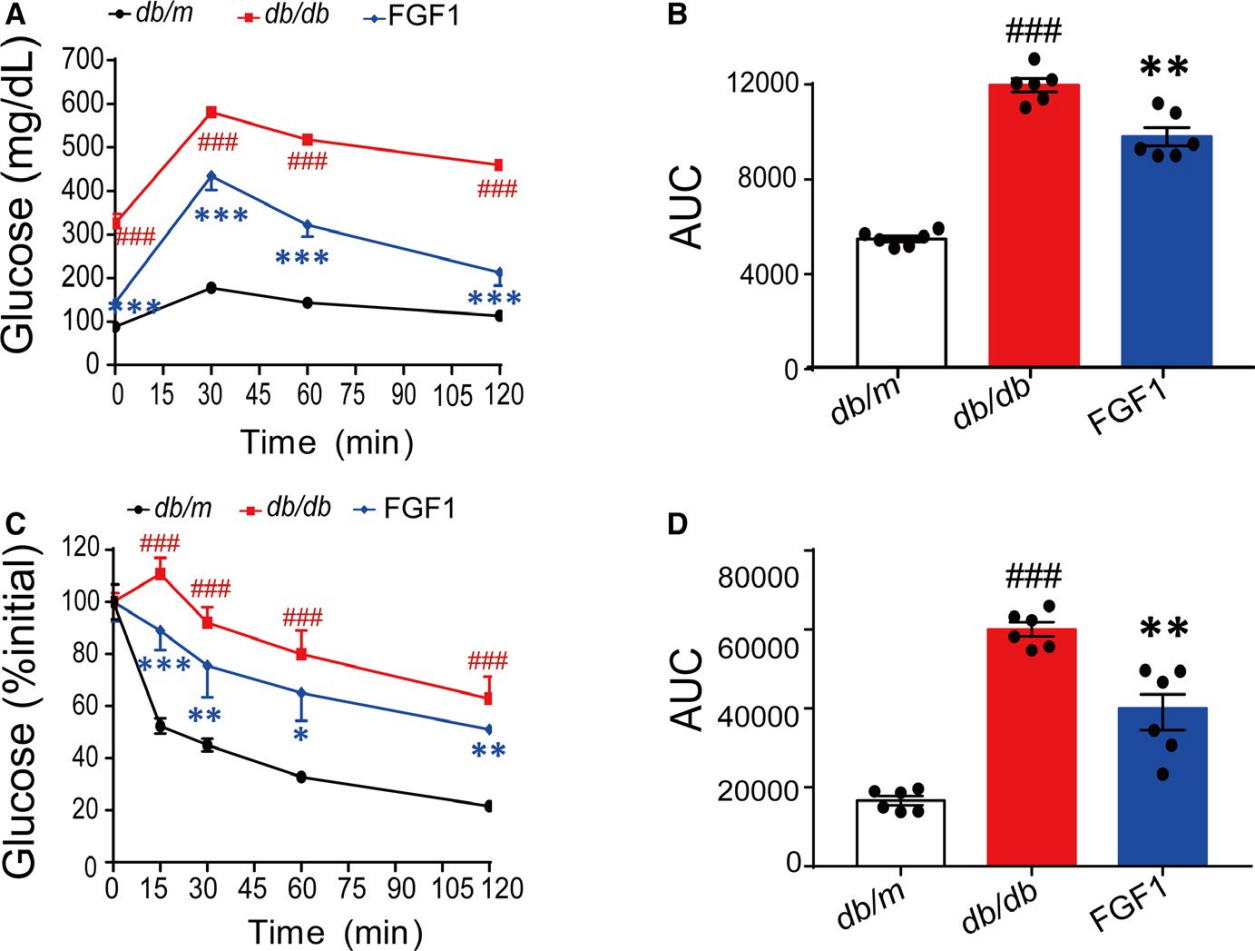
Chronic administration of FGF1 achieves the robust effect on improving insulin sensitivity in diabetic mice. A and B, Glucose tolerance test (GTT) and area under the curve of GTT (AUC) (B) of type 2 diabetic mice after chronic treatment with FGF1. C and D, Insulin tolerance test (ITT) and area under the curve of ITT (AUC) (D) of the obese mice after chronic treatment with FGF1. Data are presented as mean ± SEM (n = 6). *P < .05, **P < .01, ***P < .001, FGF1 vs *db/db*. ^#^ *P* < .05, ^##^ *P* < .01, ^###^ *P* < .001, *db/db* vs *db/m*

As a classical paracrine FGF molecule, FGF1 has been shown to function as an insulin sensitizer in blood glucose homeostasis.^20^ To evaluate the metabolic profile of FGF1 in an insulin‐resistant model, 0.5 mg/kg FGF1 was administered daily to *db/db* mice for four consecutive weeks. As shown in Figure S1A,B, FGF1 showed a robust glucose homeostasis profile, lowed the plasma insulin of *db/db* mice and did not cause hypoglycaemia and changeable plasma glucagon after chronic administration (Figure S2). Consistent with our previous report,^21^ we found that our data for the present study also indicated that the plasma glucose level of the genetically induced (*db/db*) mice remained significantly lower throughout the each time point of intraperitoneal glucose tolerance test (IPGTT) after with a prolonged administration of rhFGF1 compared with PBS treatment (Figure 1A,B). Furthermore, the IPITT results also showed that *db/db* mice treated with FGF1 had a robust improvement in insulin sensitivity compared to non‐treatment mice (Figure 1C,D).

### 3.2 FGF1 reverses obesity‐induced systemic and adipose tissue inflammatory response

**FIGURE 2:**
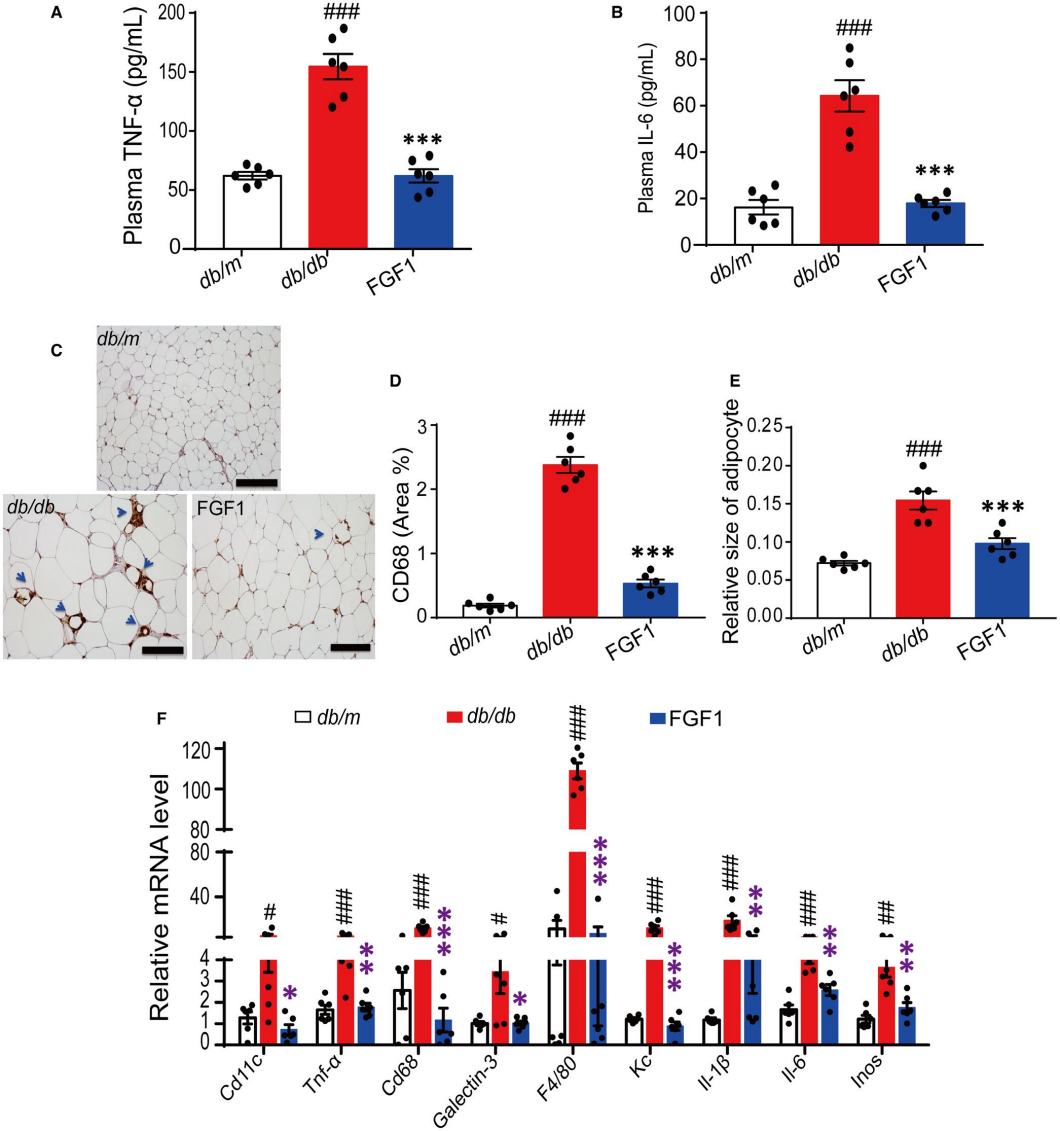
Chronic administration of FGF1 achieves the robust effect on ameliorating adipose tissue inflammation and systemic inflammation. A‐B, The plasma TNF‐α of *db/db* mice and IL‐6 level in obese mice after chronic treatment with FGF1. C‐D, The immunohistochemical staining of CD68 in epididymal adipose tissue of *db/db* mice and the quantitative analysis of positive point in tissue sections. E, The relative mRNA level of inflammatory factors in epididymal adipose tissue of *db/db* mice after chronic administration of FGF1. The data of quantitative analysis were obtained by ImageJ software. Data are presented as mean ± SEM (n = 6). *P < .05, **P < .01, ***P < .001, FGF1 vs *db/db*. ^#^ *P* < .05, ^##^ *P* < .01, ^###^ *P* < .001, *db/db* vs *db/m*

Previous reports have indicated that chronic metabolic inflammation induced systemic insulin resistance in obesity,^31^, ^32^ and these two conditions were attributed to WAT.^1^, ^2^ This study examined the potential effects of FGF1 treatment on adipose tissue and systemic inflammation in obese mice. Using enzyme linked immunosorbent assay (ELISA) kits, we assessed the serum levels of pro‐inflammatory cytokines in samples collected from the mice. The results revealed a significant reduction in the plasma levels of TNF‐α and IL‐6 in *db/db* following the administration of FGF1 (Figure 2A,B). Moreover, immunohistochemical analysis showed that the levels of CD68, an inflammatory marker, were reduced in eWAT after chronic treatment in obese mice (Figure 2C,D). This finding suggested that FGF1 had a robust effect on adipose tissue and systemic inflammation. Furthermore, in line with previous study,^19^ we found the reduced relative size of adipocyte in eWAT after chronic treatment (Figure 2E). The negative result of UCP‐1 staining suggested the chronic FGF1 treatment did not induce the beige of adipocytes in eWAT (Figure S3). Importantly, the mRNA levels of inflammatory cytokines (*Cd68*, *Tnf‐α*, *Il‐6*, *Inos*, *F4/80*, *Cd11c*, *Kc*, Il‐*1β,* *galectin‐3*) in eWAT were also significantly decreased following chronic administration of FGF1 (Figure 2F).

### 3.3 Quantitative proteome analysis reveals adipose tissue immune cells mediate the reversal of adipose tissue inflammation by FGF1

**FIGURE 3:**
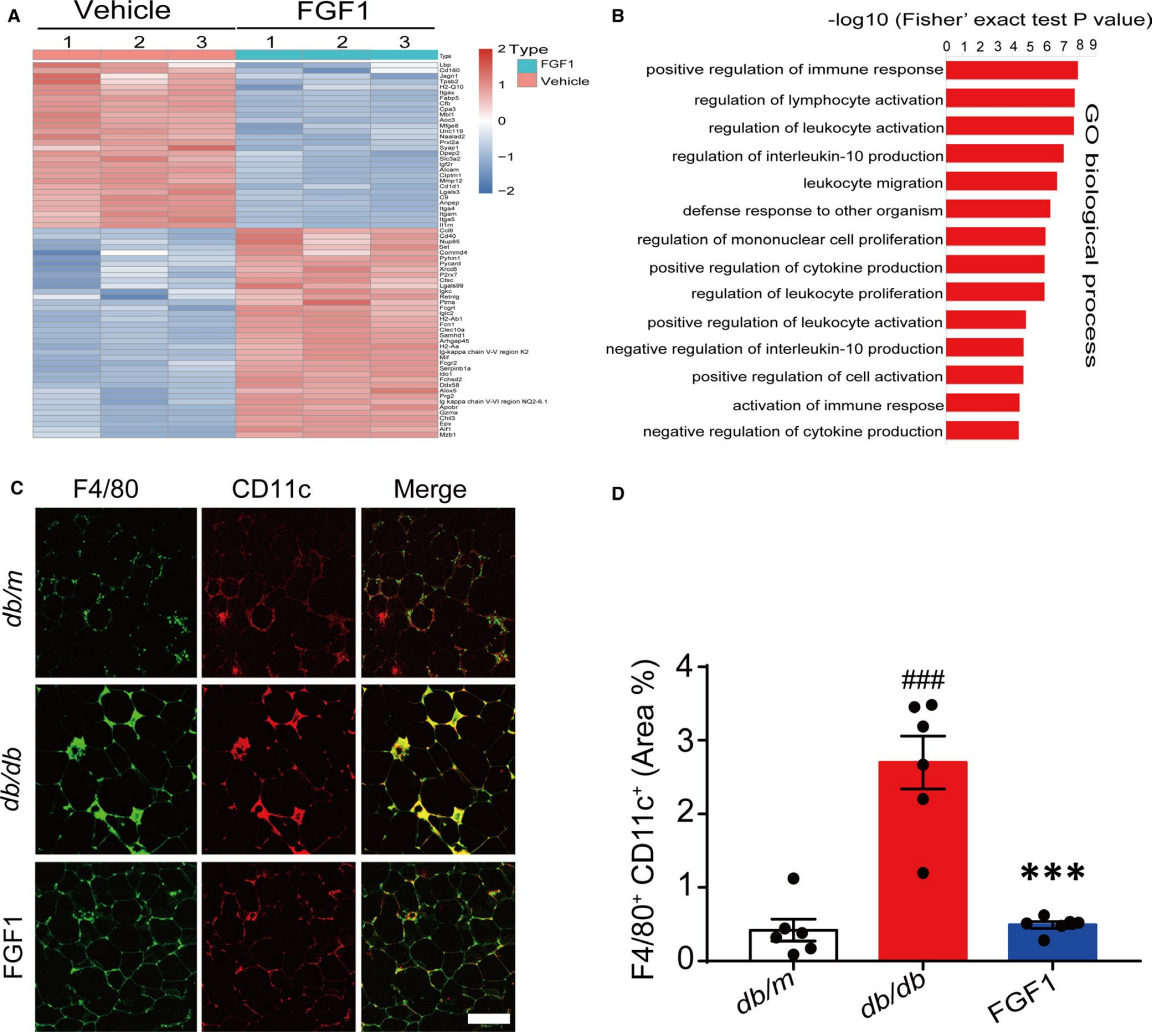
Quantitative proteome analysis reveals immune response and inflammation‐related biological process mediated the effect of FGF1 on adipose tissue inflammation. A, Cluster analysis of immune response and inflammation‐related proteins after treatment with FGF1 (Fold change = 1.5). B, GO term analysis of the regulated proteome. C‐D, Confocal merged images of epididymal fat pads from *db/db* mice, co‐stained with anti‐F4/80 (green) and CD11c (red), and the quantitative analysis data of double staining of *db/db* mice (D) were conducted by ImageJ software. Scale bar represents 500 μm for 200×; Data are presented as mean ± SEM (n = 6). *P < .05, **P < .01, ***P < .001, FGF1 vs *db/db*. ^#^ *P* < .05, ^##^ *P* < .01, ^###^ *P* < .001, *db/db* vs *db/m*

We conducted a quantitative proteome analysis of the adipose tissue to explore the mechanism by which FGF1 controlled adipose tissue inflammation. We subsequently identified approximately 5000 proteins. Under the premise of a fold change of 1.5, a total of 68 differential proteins related to adipose tissue inflammation were analysed, out of these, 38 and 30 were respectively up‐ and down‐regulated in the treatment group compared with the non‐treated controls (Figure 3A and Table S2). Furthermore, Gene Ontology (GO) analysis based on the regulated differential proteins revealed enrichment of immune cell migration, proliferation, activation and cytokine production‐related biological processes (Figure 3B), suggesting the effect of FGF1 on adipose tissue inflammation was mainly associated with immune cells migration, proliferation and related chemokines expression in eWAT.

**FIGURE 4:**
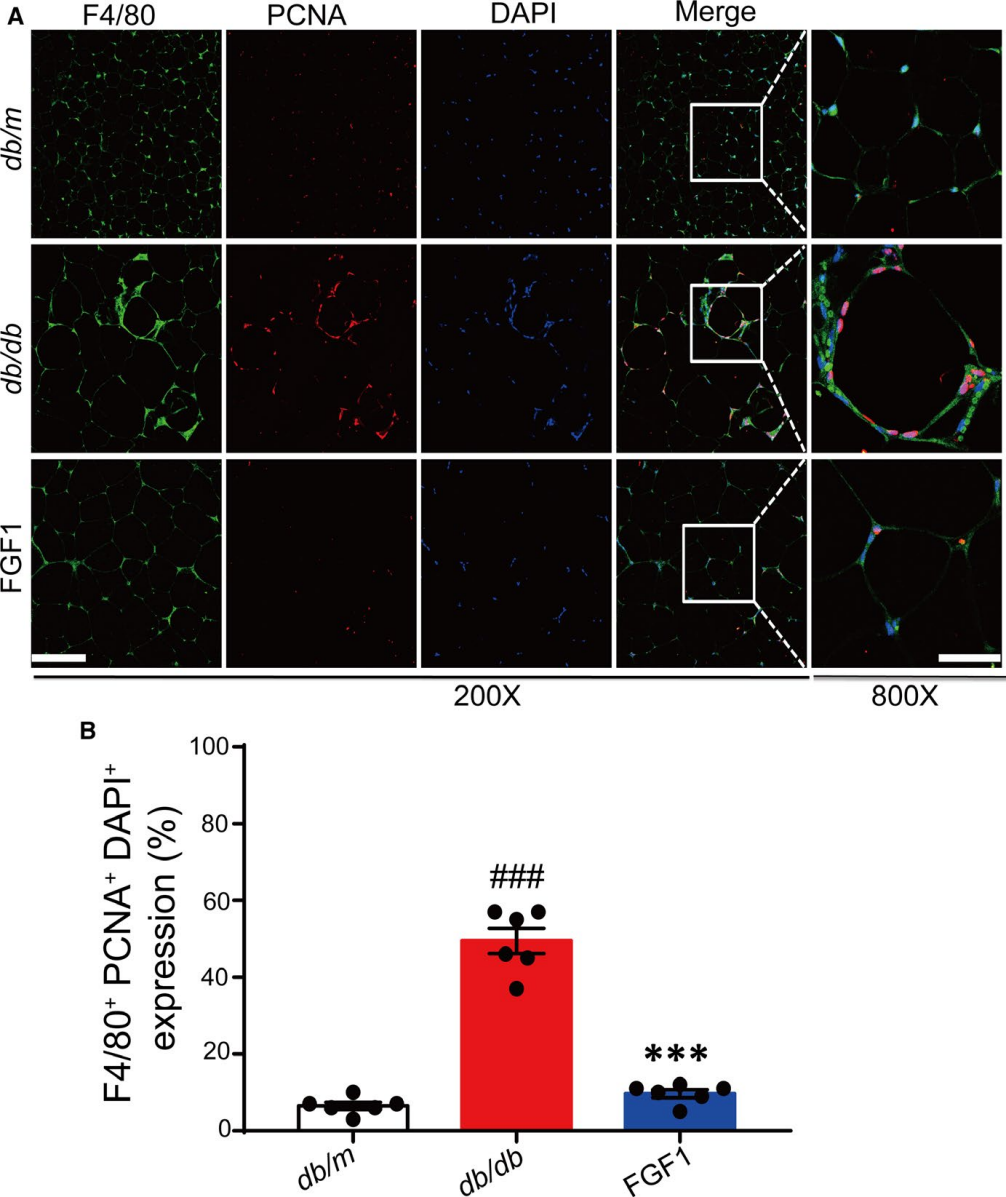
The effect of FGF1 on resident macrophage proliferation induced by obesity. A, Representative confocal merged images of epididymal adipose tissue from *db/db* mice, co‐stained with anti‐F4/80 (green) and PCNA (red), and the ImageJ software was used for quantifying the merged positive part. B, Scale bar represents 500 μm for 200× and 150 μm for 800×; Data are presented as mean ± SEM (n = 6). ***P < .001, FGF1 vs *db/db*. ^#^ *P* < .05, ^##^ *P* < .01, ^###^ *P* < .001, *db/db* vs *db/m*

To examine polarized phenotypes of pro‐inflammatory macrophages in eWAT, we conducted immunofluorescence analysis on typical macrophages markers (F4/80+ CD11c^+^). Consistent with other previous findings, our results demonstrated that *db/db* mice had a large increase in F4/80 and CD11c merged positive ATMs. These formed crown‐like structures (CLS) around adipocytes in eWAT of obese mice.^1^, ^7^ In obese mice, the number of F4/80^+^and CD11c^+^ positive ATMs exhibited a significant reduction after chronic treatment with FGF1 (Figure 3C,D). Moreover, the proliferation of resident ATMs in obese adipose tissues also decreased significantly after treatment (Figure 4A,B). Altogether, the above results suggested that FGF1 reduced the number of M1 macrophages in the eWAT of obese mice.

### 3.4 Effect of FGF1 on macrophages chemokines transcription and expression

**FIGURE 5:**
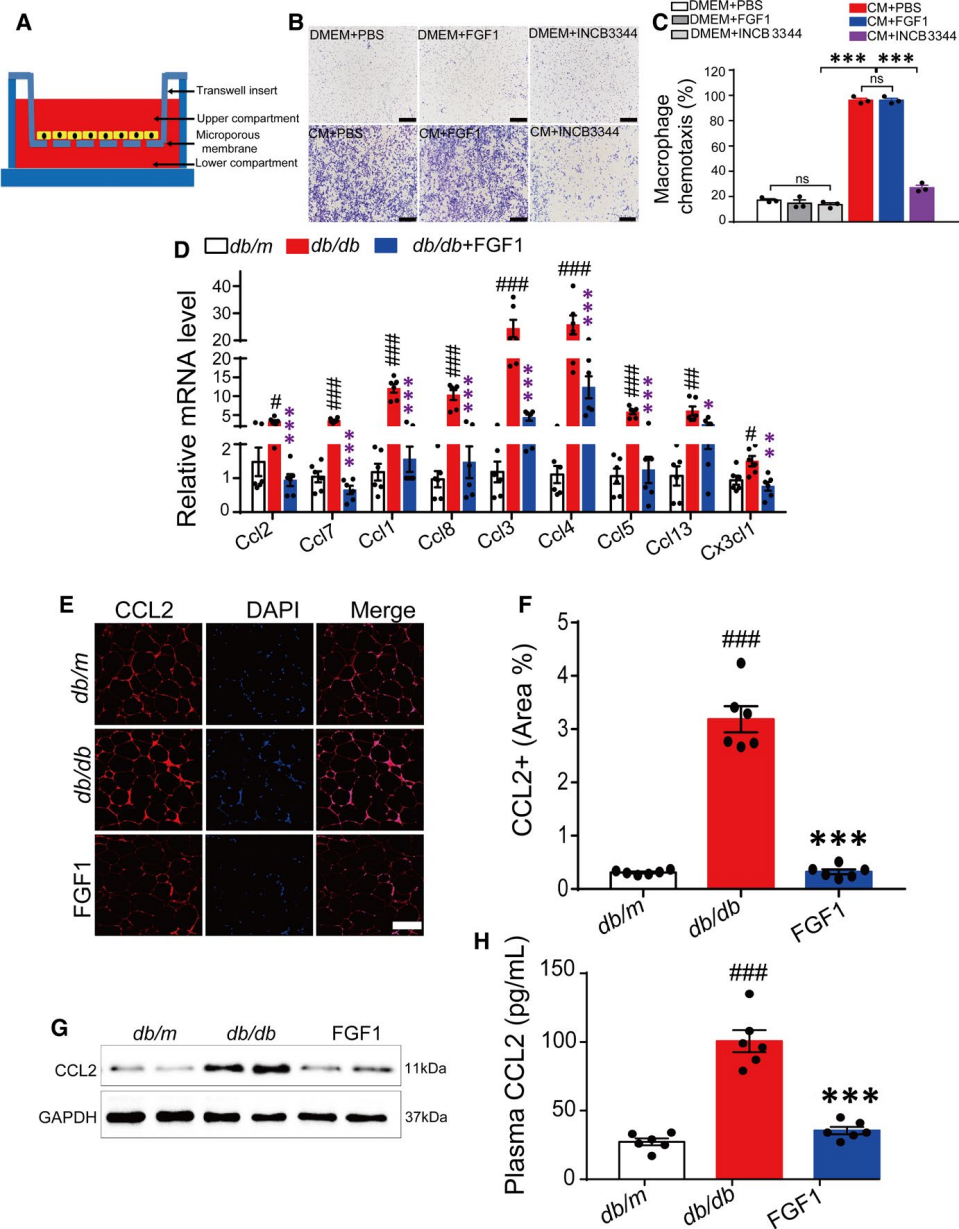
FGF1 inhibits macrophages migration by negatively controlling CCL2 transcription and expression. A, A schematic diagram showing the co‐culture of adipocytes and macrophages in vitro. B and C, The representative image of the effect of FGF1 (100 ng/mL) and INCB3344 (specific inhibitor of CCR2, 100 nmol/L) on 3T3‐L1 CM‐induced chemotaxis of macrophages (B) and the quantitative analysis (C). The image was representative of similar results from three independent experiments. Scale bar represents 1000 μm for 100×. D, The relative mRNA level of chemokines in eWAT of *db/db* mice after chronic administration of FGF1. E and F, Representative confocal merged images of CCL2 from epididymal adipose tissue of *db/db* mice, stained with anti‐CCL2 (red) and DAPI (blue), and the data of quantitative analysis of staining of *db/db* mice was conducted by ImageJ software. G, Western blot analysis of CCL2 in epididymal adipose tissue of *db/db* mice after treatment with FGF1. H, The plasma CCL2 level of *db/db* mice after treatment with FGF1. *P < .05, **P < .01, ***P < .001, FGF1 vs *db/db*. ^#^ *P* < .05, ^##^ *P* < .01, ^###^ *P* < .001, *db/db* vs *db/m*

Strategies for reducing the accumulation of ATMs have mainly focused on decreasing macrophage migration into the AT. This occurs by depleting circulating monocytes or genes encoding chemokines that attract macrophages into the AT.^13^, ^29^, ^33^, ^34^ To investigate the potential effect of FGF1 on macrophage chemotaxis and migration, we, therefore, conducted a transwell assay to evaluate whether FGF1 could inhibit macrophage migration in vitro. It is well known that a mixture of different chemokines in differentiated 3T3‐L1 adipocyte supernatant, mainly including CCL2 and other chemokines. Therefore, the conditional medium (CM) of mature 3T3‐L1 adipocyte is a typical model for evaluating macrophage chemotaxis *in vitro*. INCB3344, a specific inhibitor of CCR2 significantly blocks macrophage migration by binding CCR2.^29^ Compared with the CCR2 specific inhibitor INCB3344, FGF1 could not abolish CM‐induced chemotaxis of macrophages in the present study. This observation suggests that FGF1 cannot directly bind chemokine receptors to block macrophage migration to the conditional medium, but instead possibly regulates the transcription and expression of chemokines (Figure 5A‐C).

To verify the above‐mentioned hypothesis, we first assessed the transcription level of macrophage related chemokines by RT‐PCR. Our results demonstrated a significant decrease in chemokine levels following prolonged administration of FGF1 (Figure 5D). This confirmed that the robust effect of FGF1 on inhibiting macrophage infiltration and proliferation occurs via transcriptional regulation of chemokine expression. Given that macrophage infiltration and insulin resistance in adipose tissues is mainly mediated by the CCL2/CCR2 chemotaxis system,^13^, ^35^ we further conducted immunofluorescence and Western blot analysis to examine CCL2 protein levels in epididymal adipose tissue. In the results, treated obese mice exhibited lower expression levels of *Ccl2* compared with the model. In addition, plasma CCL2 levels decreased in obese mice after chronic treatment with FGF1 (Figure 5E‐H). Based on these findings, we suggested that the metabolic effects of FGF1 on adipose tissue inflammation and systemic insulin resistance occurred by inhibiting the transcription and expression of *Ccl2* in eWAT. The inhibition serves to block macrophage proliferation and infiltration.

### 3.5 Adipocyte mTORC2/rictor mediates the regulation of FGF1 on CCL2 transcription and expression

**FIGURE 6:**
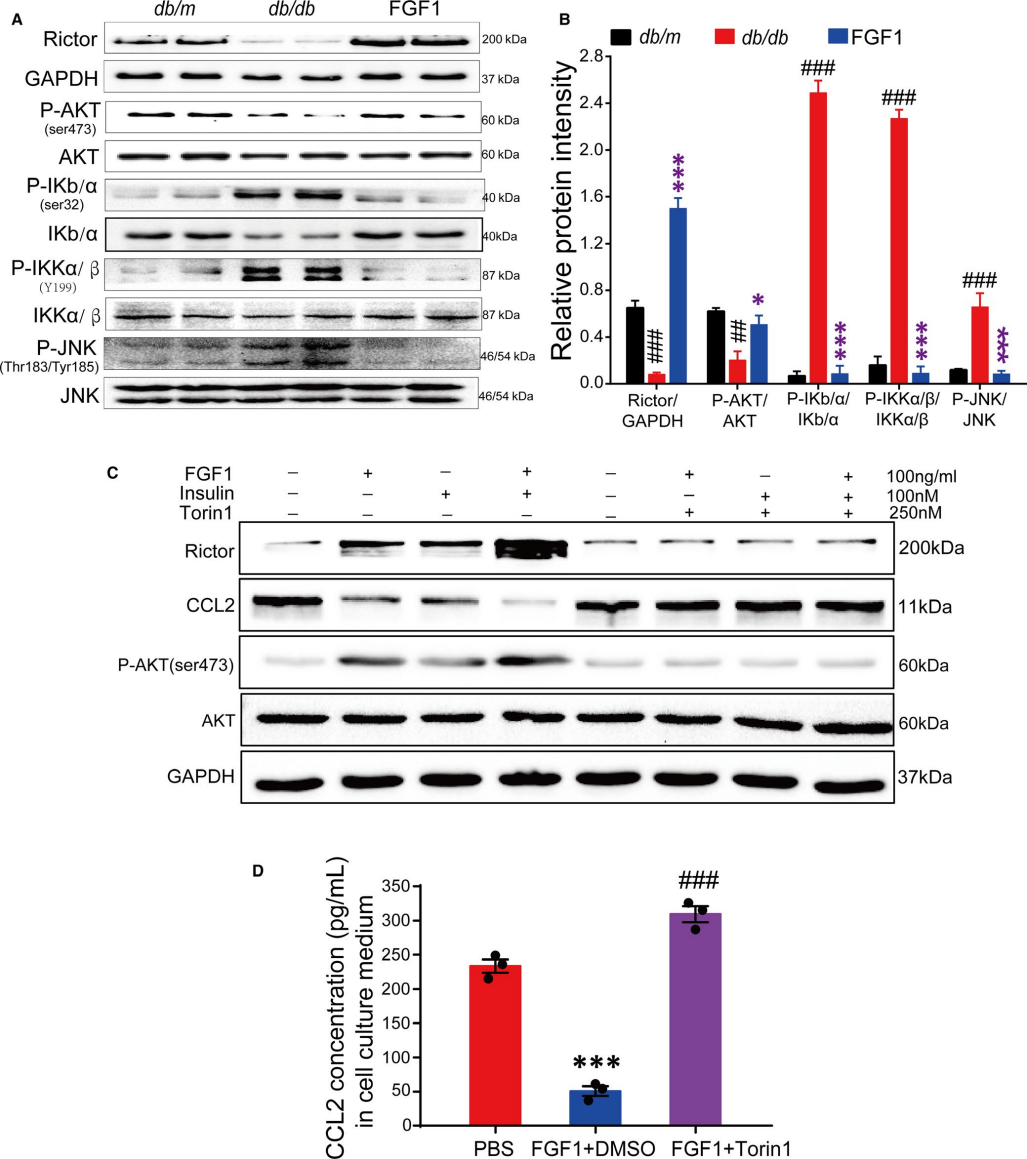
The adipocyte *mTORC2* */Rictor* mediate the effect of FGF1 on regulating Ccl2 expression. A and B, Western blot analyses of the *mTORC2/Rictor* and inflammation signalling in epididymal adipose tissue of *db/db* mice after chronic treatment with FGF1, and (B) the densitometric quantification by ImageJ software. Data are presented as mean ± SEM. *P < .05, **P < .01, ***P < .001, FGF1 vs *db/db*. ^#^ *P* < .05, ^##^ *P* < .01, ^###^ *P* < .001, *db/db* vs *db/m*. C, Western blot analysis of adipocyte rictor/mTORC2 mediate the effect of FGF1 (100 ng/mL) on regulating CCL2 production in the presence or absence of torin1 (250 nmol/L). D, The concentration of CCL2 in *3T3‐L1* conditional medial after stimulating with FGF1 in the presence or absence of torin1. Data are presented as mean ± SEM (n = 3). ***P < .001, PBS vs FGF1 + DMSO, ^###^ *P* < .001, FGF1 + Torin1 vs FGF1 + DMSO

A previous study demonstrated that in adipose tissues, *Ccl2* transcription and expression are mainly controlled by adipocyte mTORC2/Rictor via the phosphatidic acid phosphatase LIPIN1.^16^ Therefore, we confirmed the *mTORC2/* *R* *ictor* expression, AKT (Ser473) phosphorylation and readouts for mTORC2/rictor signalling in eWAT after chronic treatment with FGF1. The results showed a strong signal up of mTORC2/rictor and AKT (Ser473) phosphorylation, but a significant signal down of the inflammatory pathway, including phosphorylation of IKB/α(ser32), IKKα/β(Y199) and JNK(Thr183/Tyr185) (Figure 6A,B).

To determine whether the regulation of FGF1 on the transcription of Ccl2 was dependent on *mTORC2/Rictor*, we stimulated differentiated mature 3T3‐L1 adipocytes with FGF1 or insulin in the presence or absence of the mTOR inhibitor torin1.^36^ The 3T3‐L1 adipocytes treated with both FGF1 and insulin in the absence of torin1 exhibited a strong mTORC2/Rictor signal, as well as AKT (Ser473) phosphorylation in the adipocyte. Besides, the signal intensity was stronger compared with when either insulin or FGF1 was administered alone, thereby demonstrating synergy between the two. Conversely, the expression level of *Ccl2* significantly decreased, especially following co‐administration of FGF1 and insulin (Figure 6C,D). However, treating 3T3‐L1 adipocytes with FGF1 or insulin in the presence of torin1 revealed a reverse effect compared with treatment without torin1 (Figure 6C,D). These findings suggest that FGF1 down‐regulates the transcription and expression of *Ccl2* by enhancing adipocyte *mTORC2/Rictor* signalling. This further decreases the levels of pro‐inflammatory M1 macrophages and ameliorates adipose tissue inflammation.

### 3.6 FGF1 loses the insulin sensitizer function in AdRiKO obese mice

**FIGURE 7:**
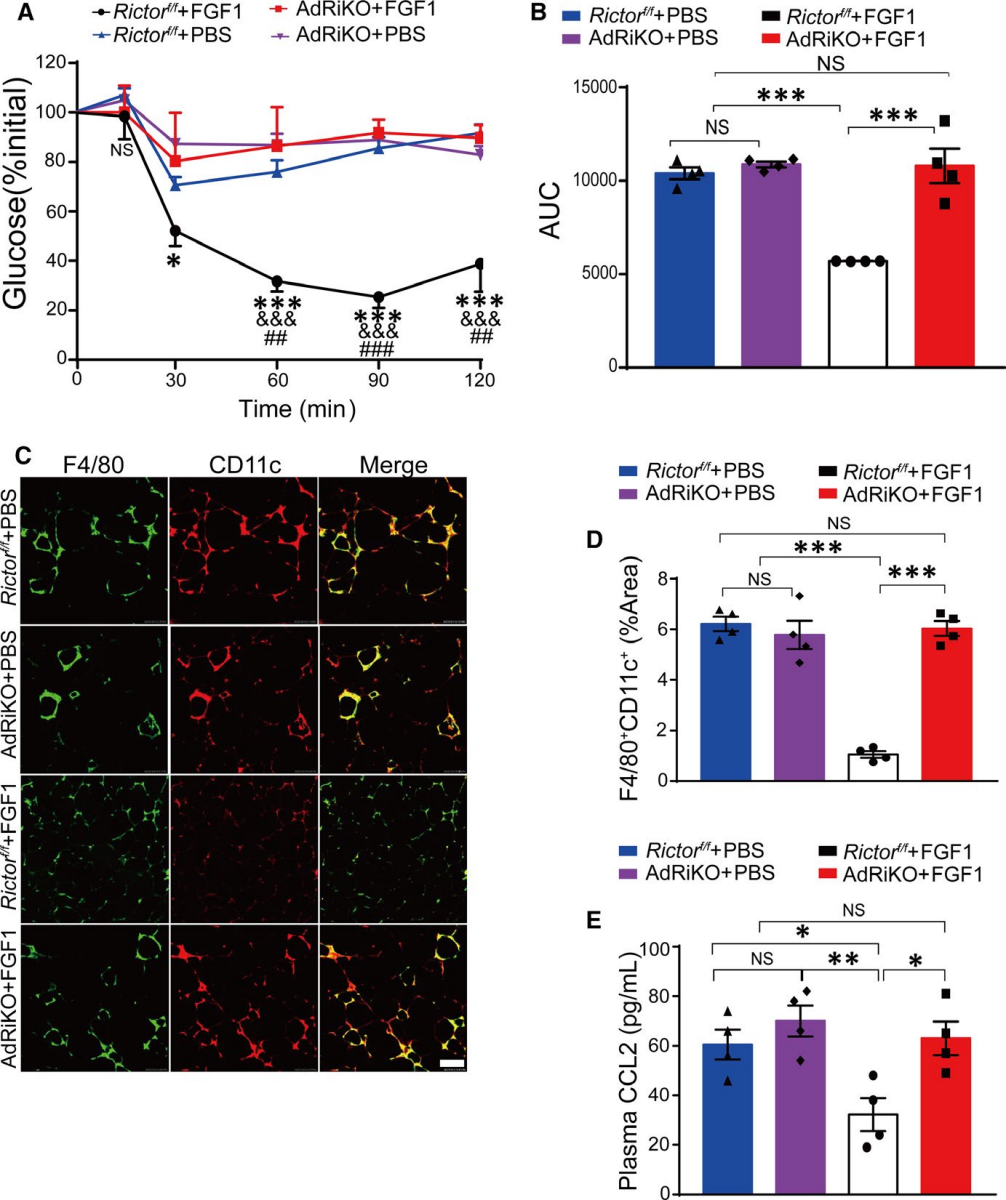
FGF1 fails to improve insulin resistance in diet‐induced AdRiKO obese mice. A, Insulin tolerance test (ITT) of AdRiKO and *R* *ictor^f/f^* obese mice after treatment with FGF1 or PBS. Data are presented as mean ± SEM (n = 4). *P < .05, **P < .01, ***P < .001, *R* *ictor^f/f^* + HFD + FGF1 vs AdRiKO + HFD + FGF1. ^&^ *P* < .05, ^&&^ *P* < .01, ^&&&^ *P* < .001, *R* *ictor^f/f^* + HFD + FGF1 vs *rictor^f/f^* + HFD + PBS. ^#^ *P* < .05, ^##^ *P* < .01, ^###^ *P* < .001, *R* *ictor^f/f^* + HFD + FGF1 vs AdRiKO + HFD + PBS. B, The area under the curve of ITT (AUC) of the AdRiKO obese mice after chronic treatment with FGF1 and PBS. Data are presented as mean ± SEM (n = 4). *P < .05, **P < .01, ***P < .001, *R* *ictor^f/f^* + HFD + FGF1 vs AdRiKO + HFD + FGF1 or AdRiKO + HFD + PBS or *R* *ictor^f/f^* + HFD + PBS. C, Representative confocal merged images of epididymal fat pads from AdRiKO and *Rictor^f/f^* fed with high‐fat diet, co‐stained with anti‐F4/80 (green) and CD11c (red), and the quantitative analysis data of double staining (D) were conducted by ImageJ software. Scale bar represents 500 μm for 200×. Data are presented as mean ± SEM (n = 4). *P < .05, **P < .01, ***P < .001, *Rictor^f/f^* + HFD + FGF1 vs AdRiKO + HFD + FGF1 or AdRiKO + HFD + PBS or *Rictor^f/f^* + HFD + PBS. E, The plasma CCL2 level of AdRiKO and *Rictor^f/f^* mice after treatment with FGF1 or PBS. Data are presented as mean ± SEM (n = 4). *P < .05, **P < .01, ***P < .001, *Rictor^f/f^* + HFD + FGF1 vs AdRiKO + HFD + FGF1 or AdRiKO + HFD + PBS or *Rictor^f/f^* + HFD + PBS

To further understand the *in vivo* role of *mTORC2/Rictor* in mediating the regulation of adipose tissue inflammation and system insulin resistance by FGF1, we generated the adipocyte‐specific *mTORC2/Rictor*‐knockout model (AdRiKO), as previously described.^16^ First, AdRiKO and *rictor^f/f^* mice were fed on HFD for 10 weeks, then treated daily with 0.5 mg/kg FGF1 for four consecutive weeks. We then conducted IPITT to evaluate the effect of FGF1 on an insulin‐resistant model of obese AdRiKO mice. Compared with the *rictor^f/f^* obese mice, obese AdRiKO mice maintained high blood glucose levels throughout the experiment. This implied that FGF1 did not ameliorate insulin resistance in obese AdRiKO mice (Figure 7A,B). Further, we used immunofluorescence and immune enzymatic reaction to assess the levels of adipose tissue macrophages and plasma CCL2. FGF1 reduced neither the number of M1 macrophages in adipose tissue nor the levels of plasma CCL2. This result could be attributed to the deletion of adipocyte *mTORC2/Rictor* (Figure 7C‐E). Altogether, the above results suggested the potential of *mTORC2/Rictor* was a crucial target of FGF1 in reversing obesity‐induced insulin resistance.

## 4 DISCUSSION

**FIGURE 8:**
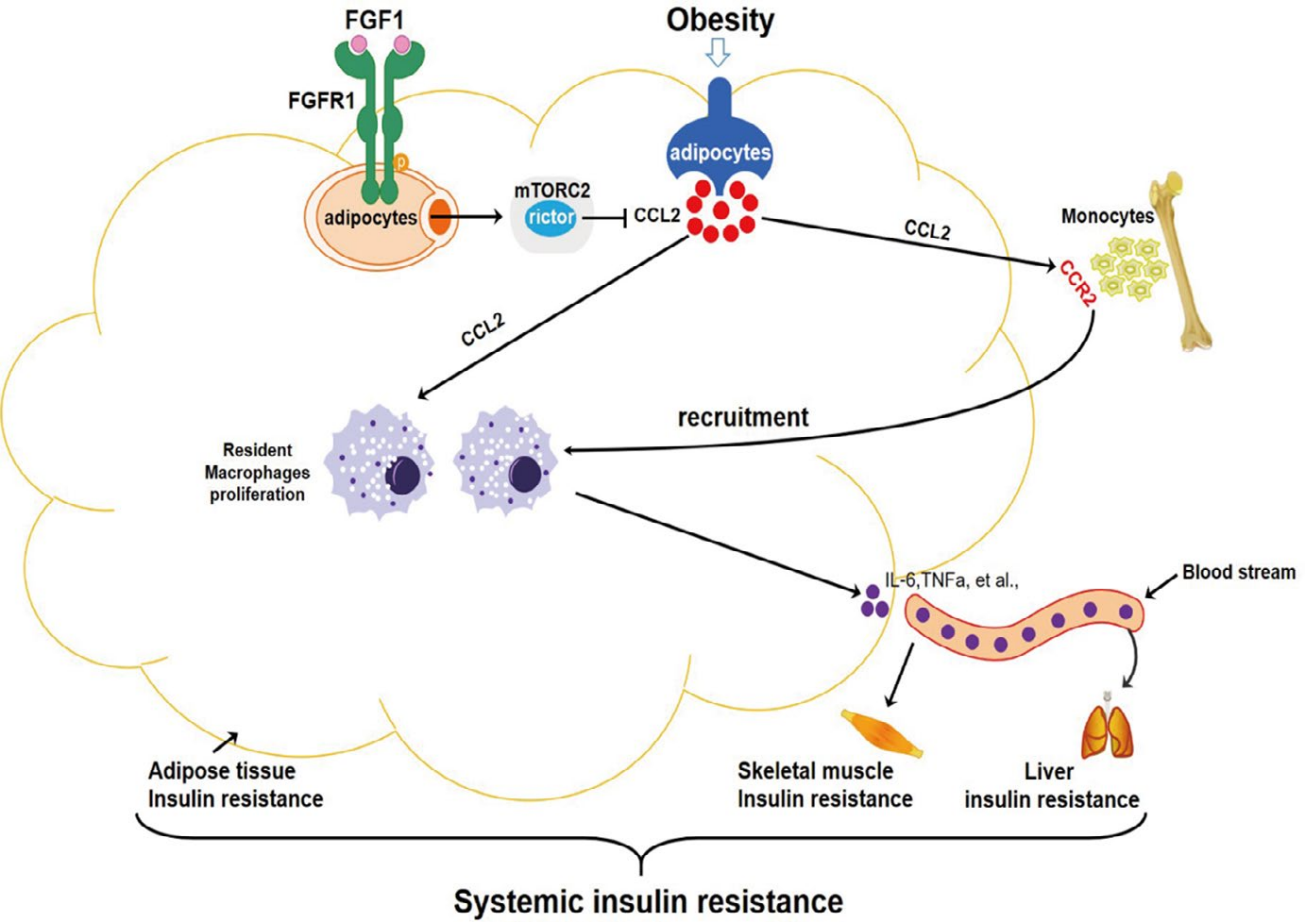
A working model of the potential protection mechanism of FGF1 against adipose tissue inflammation and systemic insulin resistance

FGF1 is a member of the paracrine FGF family known for a robust metabolic hormone function on insulin resistance and glucose homeostasis.^19^, ^20^ However, the precise FGF1 regulated signalling cascades related to obesity‐induced inflammation and systemic insulin resistance are yet to be elucidated. Besides, the underlying mechanism is obscure. In this article, we presented evidence that FGF1 ameliorated adipose tissue inflammation and insulin resistance. Specifically, we found that FGF1 significantly reduced obesity‐induced infiltration and proliferation of macrophages by enhancing the adipocyte *mTORC2/Rictor* signalling pathway. This subsequently inhibited the expression of *Ccl2* in adipose tissue, thereby ameliorating systemic insulin resistance (Figure 8).

Existing literature shows that chronic insulin resistance established in obesity is largely mediated by macrophage‐induced pro‐inflammatory responses in adipose tissues.^4^ For instance, a previous study using irradiation followed by bone marrow transplantation reported the recruitment of circulating monocytes into adipose tissue (AT) was the primary trigger for macrophage infiltration in WAT.^7^ Therefore, strategies for reducing the accumulation of ATMs mainly focus on decreasing the migration of macrophages into the AT by depleting circulating monocytes or genes encoding chemokines that attract macrophages into the AT.^13^, ^29^, ^33^, ^34^ The elevated pro‐inflammatory macrophage levels in WAT during obesity are mainly mediated by the chemokine *Ccl2*. Studies have shown CCL2 increases the number of M1 by recruiting the circulating monocytes into WATs and promoting the proliferation of resident macrophages.^9^, ^11^, ^12^ Therefore, CCL2 is the primary cause of adipose tissue inflammation. In line with the previous findings, our results indicated that FGF1 ameliorated adipose tissue inflammation and insulin resistance by regulating the transcription and expression of CCL2 (Figure 5D‐H). Although the mechanism of FGF1 for upstream regulation of *Ccl2* in adipocytes has not been elucidated yet. The adipocyte *mTORC2/Rictor* gene could be a potential target. Knocking down the adipocyte *mTORC2/Rictor* up‐regulates the expression of *Ccl2* in 3T3‐L1 adipocytes.^16^ In addition, the phosphatidic acid phosphatase LIPIN1 may modulate the effect of *mTORC2/Rictor* by controlling the production of CCL2 in adipocytes.^16^, ^37^ In the present study, we investigated the association between FGF1 and the *adipocyte mTORC2/Rictor* in regulating the expression of *Ccl2*. First, we revealed a strong signal up of *mTORC2/Rictor* and AKT (Ser473) phosphorylation. However, a significant signal down of the inflammatory pathway, including phosphorylation of IKB/α (ser32), IKKα/β (Y199) and JNK (Thr183/Tyr185), was observed in eWAT after chronic treatment with FGF1. These observations suggested that FGF1 possibly targeted *mTORC2/Rictor* signalling to inhibit *Ccl2* expression and inflammatory pathway (Figure 6A,B). We further used 3T3‐L1 mature adipocytes and *mTORC2/Rictor* inhibitor to explore the potential mechanism of the *Ccl2* regulation. Our *in vitro* results showed that FGF1 significantly enhanced *mTORC2/Rictor* signalling pathway to inhibit *Ccl2* production. Conversely, these alleviating effects of FGF1 were substantially abrogated in adipocytes with reduced *mTORC2/Rictor* expression (Figure 6C). This confirmed that *mTORC2/Rictor* was a vital target in FGF1‐induced inhibition of *Ccl2* transcription and expression.

To understand the *in vivo* role of *mTORC2/Rictor* in mediating FGF1 regulation of *Ccl2* expression, we conducted genetic‐based loss‐of‐function studies. Further, this experiment assessed the role of *mTORC2/Rictor* in mediating the effect of FGF1 on obesity‐associated adipose tissue inflammation and insulin resistance. Similar to the *in vitro* results, knocking out adipocyte *mTORC2/Rictor* led to the loss of FGF1 function in controlling *Ccl2* expression and ameliorating insulin resistance (Figure 7). In brief, our results demonstrated that FGF1 mainly inhibited macrophage infiltration by targeting the adipocyte *mTORC2/Rictor*.

Besides triggering *Ccl2* to promote the recruitment and proliferation of ATMs, adipocytes suppress the infiltration and promote alternative polarization of ATMs by secreting adiponectin.^9^, ^38^ Furthermore, other immune cells in the adipose tissue, such as T and B lymphocytes, also play a crucial role in insulin resistance. Notably, the adverse effects of adipose tissue B2 (ATB2) lymphocytes on glucose homeostasis are partly dependent on T cells and macrophages.^39^, ^40^ Other factors, such as adipocyte‐secreted exosomes, also contribute to adipose tissue inflammation by controlling the phenotypic conversion of M1/M2.^41^

Taken together, we present that FGF1 alleviates systemic insulin resistance by inhibiting macrophage recruitment and inflammatory responses in adipose tissues. Further, we demonstrate the key role of the adipocyte *mTORC2/Rictor* in mediating the effect of FGF1 on adipose tissue inflammation and systemic insulin resistance. Future studies should however explore whether other factors, such as adiponectin, T lymphocytes, B lymphocytes and exosomes, are involved in the FGF1 regulation of adipose inflammation and insulin resistance.

## CONFLICT OF INTEREST

The authors confirm that there are no conflicts of interest.

## AUTHOR CONTRIBUTIONS

**Longwei Zhao:** Conceptualization (equal); Data curation (lead); Formal analysis (lead); Investigation (equal); Methodology (equal); Writing‐original draft (lead). **Miaojuan Fan:** Data curation (equal); Methodology (equal); Software (lead); Validation (supporting). **Lijun Zhao:** Data curation (equal); Methodology (equal); Software (supporting); Validation (lead). **Yan Yang:** Conceptualization (equal); Formal analysis (equal); Investigation (lead); Project administration (supporting); Resources (equal); Writing‐original draft (supporting). **Chen Wang:** Conceptualization (equal); Formal analysis (equal); Project administration (lead); Resources (equal); Supervision (equal); Writing‐original draft (supporting). **Di Qin:** Conceptualization (lead); Formal analysis (equal); Funding acquisition (lead); Project administration (equal); Supervision (lead).

## Supporting information

App S1

## Data Availability

All data used for thisproject are publicly available and accessible online.
