# Supplementary material for: Fibroblast growth factor 1 ameliorates adipose tissue inflammation and systemic insulin resistance via enhancing adipocyte mTORC2/Rictor signal
Source: J Cell Mol Med. 2020 Sep 26;24(21):12813–25. doi: 10.1111/jcmm.15872 (PMC7687011; doi:10.1111/jcmm.15872)
Supplement: Supplementary file 1 — App S1 [file JCMM-24-12813-s001.docx]

**Supporting information**

**Journal:** [Journal of Cellular and Molecular Medicine](https://www.baidu.com/link?url=QNj1P4eoSZKFCJe7KDU44fQK3ByobCQ1Iy_-8_OUxrz3dCGufPhst96_7BOgyCGSReRcTVOru4lFFbqypA3C2w_aSV1kFuiGImteaHsc5AC&wd=&eqid=9ef6fc5100050466000000055cde0f9c).

**Title:** Fibroblast Growth Factor 1 Ameliorates Adipose Tissue Inflammation and Systemic Insulin Resistance via Enhancing Adipocyte mTORC2/Rictor Signal

**Authors:** Longwei Zhao^1^, Miaojuan Fan^1^, Lijun Zhao^4^, Yan Yang^4*^, Chen Wang^1^* and Di Qin^2,3^*

^1^ State Key Laboratory of Natural Medicines, School of Life Science and Technology, China Pharmaceutical University, 639 Longmian Avenue, Jiangning District, Nanjing 211198, China

^2^ School of Sports and Health, Nanjing Sport Institute, Nanjing, 210014, China

^3^ Jiangsu Sports and Health Engineering Collaborative Innovation Center, Nanjing, 210014, China

^4^ Maternal and Child Health Hospital of Zhuang Lang, Pingliang, 744600, PR China

*To whom correspondence should be addressed:

E-mail: sindyshu@163.com

E-mail: [cwang1971@cpu.edu.cn](mailto:cwang1971@cpu.edu.cn)

E-mail: 276378180@qq.com

**Supplementary Figure**


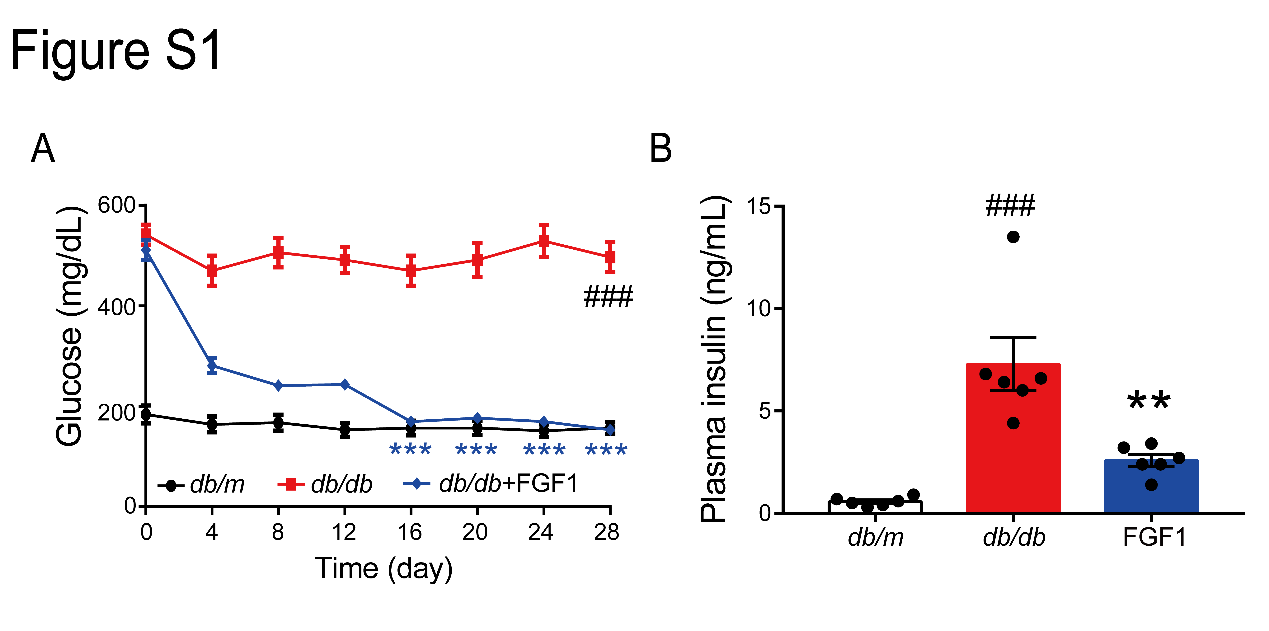


**Figure S1**: The change of type 2 diabetes glucose homeostasis and plasma insulin after chronic treatment with FGF1. **(A, B):** Blood glucose and plasma insulin levels in *db/db* mice after treatment with FGF1 and control vehicle injection. Data are presented as mean +/- SEM (n=6). *p<0.05, **p<0.01, ***p<0.001, FGF1 vs *db/db*. ^#^p<0.05, ^##^p<0.01, ^###^p<0.001, *db/db* vs *db/m*.


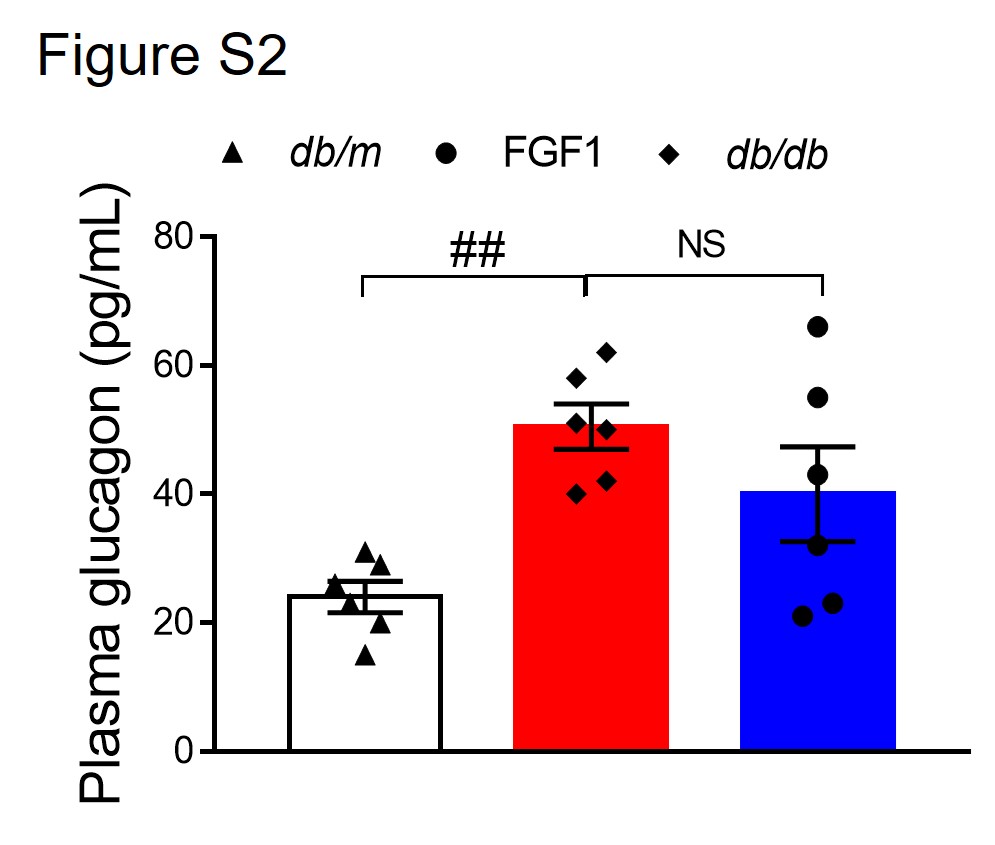


**Figure S2**: The change of type 2 diabetes plasma glucagon after chronic treatment with FGF1. Data are presented as mean +/- SEM (n=6). ^#^p<0.05, ^##^p<0.01, ^###^p<0.001, *db/db* vs *db/m*.


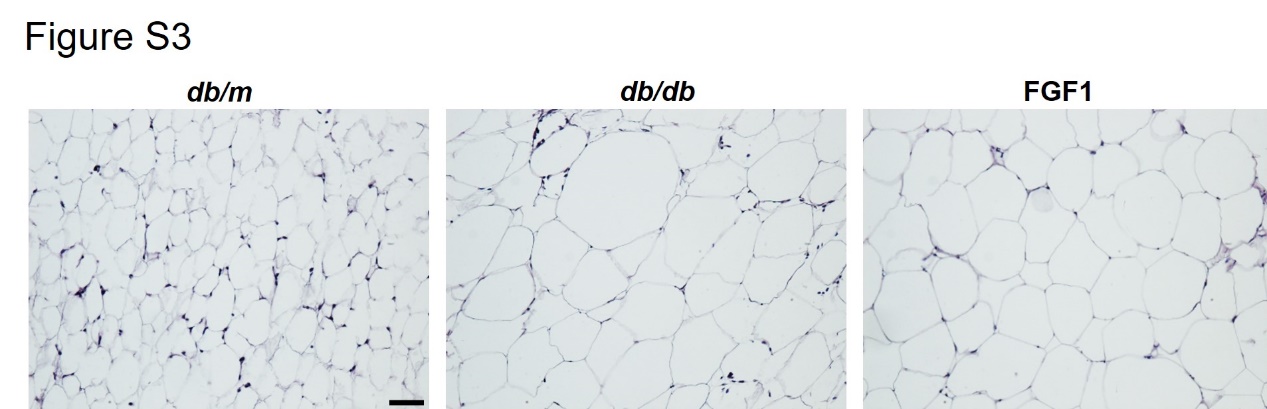


**Figure S3**: The UCP-1 staining of type 2 diabetic mice eWAT after chronic treatment with FGF1.

**Supplementary Table**

**Table S1:** qRT-PCR primers (species: mouse)

| **Target** | **Forward primer** | **Reverse primer** |
| --- | --- | --- |
| UBC | GCCCAGTGTTACCACCAAGAAG | GCTCTTTTTAGATACTGTGGTGAGGAA |
| IL-1β | GAAATGCCACCTTTTGACAGTG | TGGATGCTCTCATCAGGACAG |
| KC | ACTGCACCCAAACCGAAGTC | TGGGGACACCTTTTAGCATCTT |
| CD68 | TGTCTGATCTTGCTAGGACCG | GAGAGTAACGGCCTTTTTGTGA |
| CCL2 | TTAAAAACCTGGATCGGAACCAA | GCATTAGCTTCAGATTTACGGGT |
| F4/80 | TTGTACGTGCAACTCAGGACT | GATCCCAGAGTGTTGATGCAA |
| CD11c | CTGGATAGCCTTTCTTCTGCTG | GCACACTGTGTCCGAACTCA |
| INOS | GTTCTCAGCCCAACAATACAAGA | GTGGACGGGTCGATGTCAC |
| TNF-α | CCTGTAGCCCACGTCGTAG | GGGAGTAGACAAGGTACAACCC |
| IL-6 | TAGTCCTTCCTACCCCAATTTCC | TTGGTCCTTAGCCACTCCTTC |
| Glectin-3 | ATGAAGAACCTCCGGGAAAT | GCTTAGATCATGGCGTGGTT |
| CCL7 | CCACATGCTGCTATGTCAAGA | ACACCGACTACTGGTGATCCT |
| CCL8 | TCTACGCAGTGCTTCTTTGCC | AAGGGGGATCTTCAGCTTTAGTA |
| CCL4 | TTCCTGCTGTTTCTCTTACACCT | CTGTCTGCCTCTTTTGGTCAG |
| CCL3 | TTCTCTGTACCATGACACTCTGC | CGTGGAATCTTCCGGCTGTAG |
| CCL5 | TTCTCTGTACCATGACACTCTGC | CGTGGAATCTTCCGGCTGTAG |
| CCL1 | GGCTGCCGTGTGGATACAG | AGGTGATTTTGAACCCACGTTT |
| CXCL1 | ACGAAATGCGAAATCATGTGC | CTGTGTCGTCTCCAGGACAA |
| CCL13 | AGAAAAGATCGGCATACAAGGG | TCTCCGCGTCCATAAGATACA |
| Glectin-3 | ATGAAGAACCTCCGGGAAAT | GCTTAGATCATGGCGTGGTT |

**Table S2:** Regulated proteomes in eWAT of *db/db* and treated *db/db* mice (Related to Figure 3)

| **Gene name** | **FGF1/Vehicle Ratio** | **Regulated Type** | **FGF1/Vehicle P value** | **FGF_1** | **FGF1_2** | **FGF1_3** | **Vehicle_1** | **Vehicle_2** | **Vehicle_3** |
| --- | --- | --- | --- | --- | --- | --- | --- | --- | --- |
| --- | 1.713 | Up | 0.0020048 | 1.296 | 1.261 | 1.194 | 0.729 | 0.729 | 0.732 |
| Chil3 | 4.491 | Up | 3.6627E-05 | 1.564 | 1.58 | 1.464 | 0.387 | 0.305 | 0.334 |
| Epx | 2.752 | Up | 2.3903E-05 | 1.439 | 1.401 | 1.382 | 0.559 | 0.485 | 0.49 |
| Mzb1 | 2.566 | Up | 0.00085548 | 1.473 | 1.447 | 1.349 | 0.673 | 0.504 | 0.487 |
| Ido1 | 2.533 | Up | 5.7388E-05 | 1.31 | 1.499 | 1.434 | 0.528 | 0.556 | 0.591 |
| Fcn1 | 2.153 | Up | 1.86565E-05 | 1.409 | 1.281 | 1.347 | 0.608 | 0.642 | 0.625 |
| Retnlg | 2.06 | Up | 0.018778 | 1.403 | 1.406 | 1.2 | 0.79 | 0.43 | 0.726 |
| Prg2 | 2.051 | Up | 0.00028345 | 1.293 | 1.333 | 1.369 | 0.708 | 0.58 | 0.66 |
| Gzma | 2.029 | Up | 0.000115866 | 1.334 | 1.29 | 1.363 | 0.714 | 0.626 | 0.625 |
| Fchsd2 | 2.023 | Up | 4.35E-05 | 1.326 | 1.257 | 1.399 | 0.632 | 0.656 | 0.68 |
| Fcgr2 | 2.007 | Up | 0.00087523 | 1.301 | 1.375 | 1.293 | 0.602 | 0.613 | 0.763 |
| Pyhin1 | 1.93 | Up | 0.0171788 | 1.273 | 1.323 | 1.289 | 0.563 | 0.694 | 0.756 |
| Clec10a | 1.868 | Up | 1.70423E-05 | 1.317 | 1.253 | 1.267 | 0.689 | 0.705 | 0.66 |
| Cd40 | 1.861 | Up | 0.0024771 | 1.103 | 1.304 | 1.463 | 0.732 | 0.706 | 0.642 |
| Ccl6 | 1.839 | Up | 0.00028261 | 1.211 | 1.254 | 1.396 | 0.704 | 0.731 | 0.664 |
| Aif1 | 1.8 | Up | 0.00090062 | 1.283 | 1.212 | 1.347 | 0.798 | 0.672 | 0.664 |
| Iglc2 | 1.768 | Up | 0.00028178 | 1.324 | 1.17 | 1.333 | 0.693 | 0.747 | 0.725 |
| --- | 1.765 | Up | 0.00085997 | 1.292 | 1.211 | 1.307 | 0.772 | 0.638 | 0.749 |
| Pycard | 1.727 | Up | 0.031163 | 1.304 | 1.271 | 1.242 | 0.598 | 0.806 | 0.806 |
| Fcgrt | 1.703 | Up | 0.00198017 | 1.254 | 1.179 | 1.29 | 0.747 | 0.804 | 0.635 |
| P2rx7 | 1.688 | Up | 0.0024968 | 1.293 | 1.233 | 1.224 | 0.663 | 0.854 | 0.704 |
| Mif | 1.677 | Up | 0.00016187 | 1.269 | 1.239 | 1.159 | 0.693 | 0.757 | 0.737 |
| Set | 1.66 | Up | 0.00120492 | 1.142 | 1.307 | 1.308 | 0.692 | 0.782 | 0.789 |
| Lgals9 | 1.639 | Up | 0.0114781 | 1.197 | 1.085 | 1.368 | 0.626 | 0.855 | 0.746 |
| H2-Aa | 1.633 | Up | 0.00045579 | 1.288 | 1.307 | 1.173 | 0.727 | 0.77 | 0.811 |
| Igkc | 1.609 | Up | 0.0059232 | 1.233 | 1.151 | 1.089 | 0.716 | 0.619 | 0.824 |
| H2-Ab1 | 1.608 | Up | 0.00025705 | 1.266 | 1.182 | 1.215 | 0.724 | 0.809 | 0.745 |
| Ctsc | 1.581 | Up | 0.0034021 | 1.313 | 1.148 | 1.271 | 0.693 | 0.845 | 0.822 |
| Xrcc6 | 1.559 | Up | 0.0081997 | 1.311 | 1.173 | 1.163 | 0.671 | 0.897 | 0.771 |
| Arhgap45 | 1.558 | Up | 4.1353E-05 | 1.252 | 1.204 | 1.178 | 0.764 | 0.8 | 0.768 |
| Samhd1 | 1.556 | Up | 1.62672E-05 | 1.242 | 1.212 | 1.189 | 0.774 | 0.798 | 0.77 |
| Serpinb1a | 1.551 | Up | 0.0062845 | 1.211 | 1.23 | 1.209 | 0.738 | 0.784 | 0.832 |
| Ptms | 1.548 | Up | 0.021736 | 1.376 | 1.135 | 1.131 | 0.79 | 0.78 | 0.782 |
| Nup85 | 1.523 | Up | 0.0060244 | 1.073 | 1.195 | 1.341 | 0.738 | 0.86 | 0.771 |
| Alox5 | 1.515 | Up | 0.0020226 | 1.155 | 1.283 | 1.128 | 0.838 | 0.724 | 0.792 |
| Commd4 | 1.513 | Up | 0.0448 | 1.061 | 1.231 | 1.304 | 0.607 | 0.966 | 0.804 |
| Apobr | 1.51 | Up | 0.000158349 | 1.16 | 1.246 | 1.219 | 0.834 | 0.782 | 0.785 |
| Ddx58 | 1.503 | Up | 1.72007E-05 | 1.179 | 1.195 | 1.232 | 0.808 | 0.785 | 0.807 |
| Il1rn | 0.666 | Down | 8.3416E-05 | 0.809 | 0.823 | 0.846 | 1.187 | 1.27 | 1.262 |
| Mfge8 | 0.665 | Down | 0.00130347 | 0.856 | 0.868 | 0.748 | 1.284 | 1.23 | 1.204 |
| Fabp5 | 0.659 | Down | 1.5658E-06 | 0.822 | 0.794 | 0.802 | 1.223 | 1.233 | 1.214 |
| H2-Q10 | 0.656 | Down | 0.0064038 | 0.864 | 0.823 | 0.679 | 1.266 | 1.124 | 1.216 |
| Clptm1 | 0.654 | Down | 2.048E-05 | 0.778 | 0.797 | 0.809 | 1.202 | 1.25 | 1.193 |
| Syap1 | 0.652 | Down | 0.004403 | 0.802 | 0.805 | 0.743 | 1.076 | 1.168 | 1.358 |
| Mmp12 | 0.64 | Down | 0.000101911 | 0.771 | 0.786 | 0.824 | 1.26 | 1.273 | 1.189 |
| Lbp | 0.638 | Down | 0.027364 | 0.733 | 0.941 | 0.692 | 1.417 | 1.264 | 1.03 |
| Cpa3 | 0.63 | Down | 7.4651E-07 | 0.796 | 0.778 | 0.786 | 1.264 | 1.23 | 1.25 |
| Itgam | 0.626 | Down | 0.0091386 | 0.755 | 0.761 | 0.765 | 1.11 | 1.283 | 1.252 |
| Lgals3 | 0.624 | Down | 0.00023509 | 0.758 | 0.769 | 0.817 | 1.203 | 1.226 | 1.325 |
| Dpep2 | 0.605 | Down | 0.00079966 | 0.759 | 0.701 | 0.843 | 1.306 | 1.253 | 1.248 |
| Cd1d1 | 0.589 | Down | 0.00040389 | 0.838 | 0.732 | 0.743 | 1.25 | 1.326 | 1.352 |
| Unc119 | 0.589 | Down | 0.00119911 | 0.76 | 0.834 | 0.674 | 1.286 | 1.325 | 1.237 |
| Slc3a2 | 0.585 | Down | 0.00059892 | 0.741 | 0.728 | 0.817 | 1.268 | 1.41 | 1.232 |
| Cfb | 0.582 | Down | 4.006E-06 | 0.738 | 0.74 | 0.715 | 1.282 | 1.22 | 1.264 |
| Prxl2a | 0.569 | Down | 0.0027596 | 0.709 | 0.783 | 0.728 | 1.304 | 1.296 | 1.303 |
| Igf2r | 0.567 | Down | 2.0919E-05 | 0.715 | 0.708 | 0.765 | 1.297 | 1.288 | 1.274 |
| Tpsb2 | 0.558 | Down | 0.0055599 | 0.774 | 0.732 | 0.691 | 1.555 | 1.099 | 1.282 |
| Mbl1 | 0.556 | Down | 0.00042053 | 0.738 | 0.733 | 0.687 | 1.424 | 1.224 | 1.233 |
| Naalad2 | 0.553 | Down | 0.00098349 | 0.674 | 0.791 | 0.715 | 1.443 | 1.285 | 1.217 |
| Itgax | 0.541 | Down | 0.000158505 | 0.748 | 0.694 | 0.733 | 1.411 | 1.243 | 1.369 |
| Aoc3 | 0.535 | Down | 0.000119882 | 0.661 | 0.68 | 0.71 | 1.373 | 1.239 | 1.223 |
| Itga5 | 0.516 | Down | 0.00024302 | 0.68 | 0.698 | 0.708 | 1.221 | 1.358 | 1.46 |
| Jagn1 | 0.493 | Down | 0.0153229 | 0.731 | 0.55 | 0.748 | 1.706 | 1.049 | 1.364 |
| Alcam | 0.449 | Down | 7.6805E-05 | 0.617 | 0.6 | 0.696 | 1.381 | 1.449 | 1.435 |
| Cd180 | 0.418 | Down | 0.0154378 | 0.394 | 0.853 | 0.566 | 1.467 | 1.534 | 1.334 |
| Anpep | 0.399 | Down | 5.752E-05 | 0.618 | 0.553 | 0.593 | 1.361 | 1.545 | 1.51 |
| Itga4 | 0.379 | Down | 5.9519E-05 | 0.567 | 0.528 | 0.599 | 1.373 | 1.529 | 1.567 |
| C9 | 0.359 | Down | 0.00032103 | 0.547 | 0.49 | 0.592 | 1.319 | 1.67 | 1.551 |

**Supplementary Materials and Methods**

**Proteome analysis (This method was provided by PTM-Biolabs Co., Ltd. Hangzhou, China)**

eWAT was isolated from *db/db* and *db/db* mice treated with FGF1. Protein was extracted from isolated eWAT in ice-cold lysis buffer (8 M urea, 2 mM EDTA, 10 mM DTT and 1% Protease Inhibitor Cocktail III) using a high-intensity ultrasonic processor (SCIENTZ Biotechnology, Ningbo, China) on ice. The remaining debris were removed by centrifugation at 20,000 ×g at 4 °C for 10 min. Finally, the protein was precipitated with cold 15% Trichloroacetic for 2 h at -20 °C. After centrifugation at 4 °C for 10 min, the supernatant was discarded. The remaining precipitate was washed with cold acetone three times. The protein was dissolved in buffer (8 M urea, 100 mM TEAB, pH 8.0), and the protein concentration was determined with a 2-D Quant kit according to the manufacturer’s instructions. The aliquots were stored at -80 °C for further proteomic and Western blotting studies. The pooling of animal samples is a cost-effective approach for proteomic studies; therefore, we selected 3 *db/db mice* (vehicle-1, vehicle-2, vehicle-3) and 3 treated *db/db* *mice* (FGF1-1, FGF1-2, FGF1-3).

For trypsin digestion, the protein solution was reduced with 10 mM DTT for 1 h at 37 °C and alkylated with 20 mM IAA for 45 min at room temperature in the dark. Then, the protein sample was diluted by adding 100 mM TEAB until the urea concentration was less than 2 M. Finally, trypsin was added at a 1:50 trypsin-to-protein mass ratio for the first digestion overnight and a 1:100 trypsin-to-protein mass ratio for a second 4 h digestion. Approximately 100 μg of protein for each sample was digested with trypsin for the following experiments [1]. After trypsin digestion, peptides were desalted using a Strata X C18 SPE column (Phenomenex, Torrance, CA) and vacuum-dried. The peptides were reconstituted in 0.5 M TEAB and processed according to the manufacturer’s protocol for the 6-plex TMT kit. Briefly, one unit of TMT reagent (defined as the amount of reagent required to label 100 μg of protein) was thawed and reconstituted in 24 μl of ACN. The peptide mixtures were then incubated for 2 h at room temperature and pooled, desalted and dried by vacuum centrifugation. The sample was then fractionated by high pH reverse-phase HPLC using an Agilent 300Extend C18 column (5 μm particles, 4.6 mm ID, 250 mm in length). Briefly, the peptides were first separated with a gradient of 2% to 60% acetonitrile in 10 mM ammonium bicarbonate pH 10 over 80 min into 80 fractions. Then, the peptides were combined into 18 fractions and dried by vacuum centrifugation.

Peptides dissolved in 0.1% FA were directly loaded onto a reversed-phase pre-column (Acclaim PepMap 100, Thermo Scientific). Peptide separation was performed with a reversed-phase analytical column (Acclaim PepMap RSLC, Thermo Scientific). The gradient consisted of an increase from 6% to 22% solvent B (0.1% FA in 98% ACN) over 26 min, 22% to 35% in 8 min and 35% to 80% in 3 min, then holding at 80% for the last 3 min, all at a constant flow rate of 400 nl/min on an EASY-nLC 1000 UPLC system (Thermo Scientific). The resulting peptides were analyzed by a Q Exactive Plus Hybrid Quadrupole-Orbitrap mass spectrometer (Thermo Scientific). The peptides were subjected to nanospray-ionization followed by tandem mass spectrometry (MS/MS) in a Q Exactive Plus (Thermo Scientific) coupled online to the UPLC. Intact peptides were detected in the Orbitrap at a resolution of 70,000. Peptides were selected for MS/MS using a normalized collision energy set at 30; ion fragments were detected in the Orbitrap at a resolution of 17,500. A data-dependent procedure that alternated between one MS scan followed by 20 MS/MS scans was applied for the top 20 precursor ions above a threshold ion count of 10,000 in the MS survey scan with 30.0 s dynamic exclusion. The electrospray voltage applied was 2.0 kV. Automatic gain control was used to prevent overfilling of the Orbitrap; 50,000 ions were accumulated to generate MS/MS spectra. For MS scans, the m/z scan range was 350 to 1800. The fixed first mass was set as 100 m/z. The resulting MS/MS data were processed using the Mascot search engine (v.2.3.0). Tandem mass spectra were searched against the Swiss Prot Human database. Trypsin/P was specified as the cleavage enzyme, allowing up to 2 missing cleavages. The mass error was set to 10 ppm for precursor ions and 0.02 Da for-fragment ions. Carbamidomethylation of cysteine residues was specified as a fixed modification, and methionine oxidation was specified as a variable modification. For the protein quantification method, TMT-6-plex was selected in Mascot. The FDR was adjusted to < 1%, and the peptide ion score was set at ≥ 20 [2].

1. **Tsai TH, Song E, Zhu R, Di Poto C, Wang M, Luo Y, Varghese RS, Tadesse MG, Ziada DH, Desai CS, Shetty K, Mechref Y, Ressom HW.** LC-MS/MS-based serum proteomics for identification of candidate biomarkers for hepatocellular carcinoma. *Proteomics*. 2015; 15: 2369-81.

2. **Li F, Wang Y, Li Y, Yang H, Wang H.** Quantitative Analysis of the Global Proteome in Peripheral Blood Mononuclear Cells from Patients with New-Onset Psoriasis. *Proteomics*. 2018; 18: e1800003.
